# Factors Affecting Australian Catholics’ Return to Mass After COVID-19 Church Closures

**DOI:** 10.1007/s10943-022-01618-1

**Published:** 2022-07-28

**Authors:** Philippa Martyr

**Affiliations:** 1 School of Psychological Sciences, Monash University, Victoria, Australia; 2 School of Biomedical Sciences, University of Western Australia, Perth, WA Australia

**Keywords:** COVID-19, Catholic, Australia, Mass attendance, Virtual worship

## Abstract

In 2020, many places of worship closed due to COVID-19 restrictions, raising questions about rates of return to worship after COVID-19. This survey-based study of 806 Australian churchgoing Catholics explores relationships between a range of variables and the rate of return to Mass attendance after church closures. Pre-closure Mass attendance rate strongly and significantly predicted real-life worship during church closures and higher rates of return to Mass attendance after churches reopened. Real-life worship during COVID-19 also strongly predicted return to Mass attendance, and positively mediated the relationship between pre- and post-closure Mass attendance rates. Virtual worship engagement did not significantly predict return to Mass attendance, but positively mediated the relationship between pre- and post-closure Mass attendance rates, with a smaller effect size.

## Introduction

The closure of places of worship due to COVID-19 restrictions has affected many religious traditions such as Judaism (Langer, 2021), Islam, and Catholicism (El‑Majzoub et al., 2021). Catholic sacramental worship depends on face-to-face, in-person ritual contact (Sarah, 2020; Schmalzbauer, 2006). For example, confessions cannot be heard over the phone or Internet: the priest and the person confessing must be physically present to one another (Flynn & Condon, 2020). Because of this, virtual forms of Catholic worship—televised, livestreamed, or online—are not customary or popular, although Mass was first televised in 1948 (John Paul II, 1998).

Given this, mandated church closures during COVID-19 could be expected to have an adverse effect on churchgoing Catholic communities (Baker et al., 2020). Sunday Mass attendance rates in most Western countries have fallen since the 1950s (Berman et al., 2018; Schwadel, 2010). In Australia the rate has fallen from around 74% of all Catholics in Australia in the 1950s to around 11% in 2016 (Cave & Albeck-Ripka, 2019; National Centre for Pastoral Research [NCPR], 2013, 2020). There are currently around 600,000 Australian churchgoing Catholics (those who have been baptised or otherwise received into the Catholic Church and who attend Sunday and other mandatory Masses, Stock, 2009). In this study, a ‘churchgoing Catholic’ was defined as someone who would attend Mass at least several times a month. This group shows a higher level of affiliation and ongoing engagement with public worship than those Catholics who normally attend church only at Christmas and Easter, or for family occasions. Recent data show that the churchgoing Catholic population in Australia is mostly aged over 50 years, is around two-thirds female, and is highly urbanised (NCPR, 2020). With already falling rates of Mass attendance, the longer-term impact of COVID-19 church closures is likely to be significant in determining the future size and distribution of the Catholic Church in Australia.

To prevent the spread of COVID-19, places of worship across Australia began closing from early 2020. Some Catholic churches voluntarily ceased public worship by 19 March 2020 (Archdiocese of Perth, 2020), and all Catholic churches and chapels in Australia were closed by federal government order on 23 March 2020 (Rodrigues, 2020a). Churches in most parts of Australia began to reopen from June 2020, except for the state of Victoria, which maintained longer lockdowns.

Australian Catholic bishops conformed to both State and federal government restrictions: all 28 dioceses, plus the five Eastern Rite eparchies and two ordinariates, ceased public celebration of Mass, and restricted the number of attendees at baptisms, weddings, and funerals (Diocese of Armidale, 2020; Diocese of Sale, 2020). Catholics were dispensed from their obligation to attend Sunday Mass—that is, given formal permission by their bishop not to attend—and were encouraged to pray at home and watch televised and online livestreamed church services (Cramsie, 2020; Rosengren, 2020). Most dioceses encouraged priests to say Mass by themselves without others present, and to minister to the sick and elderly with appropriate social distancing and personal protective equipment (PPE) (Archdiocese of Melbourne, 2020; Diocese of Wagga Wagga, 2020).

During church closures, however, it was still possible for some Catholics to participate in real-life worship. Catholics can receive sacraments individually: for example, receiving Holy Communion from another person without attending a Mass (International Commission on the Liturgy, 1976). Only three dioceses in Australia suspended this individual practice altogether (Diocese of Wollongong, 2020; Archdiocese of Canberra-Goulburn, 2020; Diocese of Wagga Wagga, 2020). Similarly, only three dioceses suspended the hearing of individual confessions by priests (Archdiocese of Brisbane, 2020; Diocese of Cairns, 2020; Diocese of Wollongong, 2020). In most parts of Australia during the 2020 church closures, a priest could distribute Holy Communion to individuals or hear individual confessions with social distancing and PPE, without breaching any Australian public health regulations or diocesan guidelines. Up to ten technical staff—usually lay people—were permitted to assist with livestreaming Catholic Masses and receive Holy Communion there as well (Archdiocese of Melbourne, 2020; Hennessey, 2020).

Catholic media sources and spokespersons described church closures as unjust while retail outlets like hairdressing salons remained open or re-opened (CNA, 2020; Comensoli, 2020; Rodrigues, 2020b). Some expressed anger at what they saw as capitulation to secular authorities by bishops (Donnelly, 2020). As places of worship began to reopen, some church leaders expressed concern over the impact of closures on their communities’ mental health and wellbeing, and whether Catholics would return to Mass once churches reopened (Goulding, 2020; Patterson, 2020).

There are relatively few pre-COVID-19 studies of Christian virtual versus real-life worship. However, studies undertaken in Anglican, Lutheran, and evangelical communities have consistently found that the experience of televised or digital worship was unsatisfying (Wolff, 1999; Hutchings, 2011; Campbell & DeLashmutt, 2014; Grayson, 2017). Specifically, virtual worship lacked a distinctive separation from everyday life, blurred the boundaries between spectator entertainment and religion, and was not experienced as valid, transformative, renewing, or immersive.

These studies all examined experimental online communities where participants made a free choice between virtual or real-life worship. However, during COVID-19 virtual worship was imposed rather than chosen. The first UK-based survey of churchgoing Catholics during the COVID-19 general lockdown (*N* = 2292) found lower rates of virtual worship engagement among Catholics, compared to non-Catholics. Most Catholic respondents also showed a strong preference for real-life worship, with only 4% indicating that they would use exclusively virtual worship in future (CatholicVoices, 2020). A follow-up survey in 2021 confirmed these results, with higher rates of Catholic participants feeling more fulfilled and less distracted with real-life worship attendance, compared to online worship (*N* = 1018). Similarly, only 6% agreed that virtual contact is as good as face-to-face, and 85% agreed that local churches, rather than online churches, should be maintained (Village, Francis, & Davis, 2021).

There is currently little research literature exploring the impact of church closures of COVID-19 on Catholics in Australia (Fleming & McIlroy, 2021; McCarthy, 2021; Tan et al., 2021). Prior to COVID-19, Dunne et al. (1997) found in a sample of 1631 adult Australian churchgoing Catholics that frequent church attendance was related to lower neuroticism and psychoticism scores with a modest effect size. The National Church Life Survey’s 2011 data set generated several studies of adult churchgoing Christians and psychological wellbeing in Australia, including some Catholic data, but found few differences between Catholics and Protestants (Francis et al., 2016; Powell & Pepper, 2015; Powell & Robbins, 2015).

Given the real-life orientation of most forms of Catholic worship, Australian churchgoing Catholics may have been dissatisfied with virtual worship as a substitute for real-life worship. For very frequent Mass attenders (attending Mass more than once a week), real-life worship opportunities during church closures may have been more appealing and accessed more frequently than virtual worship opportunities. There may also be a relationship between the rate of accessing real-life worship during church closures and rate of return to Mass attendance once churches reopen. Hypothetically there may be a cascade effect: higher rates of Mass attendance prior to church closures may predispose churchgoing Catholics to prefer real-life to virtual worship when churches are closed, and this in turn may have had a protective effect on Mass attendance rates once churches re-opened.

The aim of this study is to explore possible relationships between demographic factors (age and gender), real-life and virtual worship engagement during church closures, and pre-and post-closure Mass attendance rates in an Australian Catholic churchgoing population. The hypotheses are:

- That the rate of pre-closure Mass attendance is significantly and positively related to the rate of engagement in real-life worship during church closures.
- That the rate of pre-closure Mass attendance is not significantly related to the rate of engagement in virtual worship during church closures.
- That the rate of engagement in real-life worship during church closures is significantly and positively related to the rate of return to Mass attendance after closures.
- That the rate of engagement in real-life worship during church closures positively mediates the relationship between pre-closure and post-closure rates of Mass attendance.

## Method

As part of a national study on Catholic worship choices during COVID-19, a convenience sample of 1173 adult participants (39.3% males, 59.8% females, 0.9% other) was recruited nationally from 22 August–5 October 2020 via Australian Catholic networks, including Facebook groups, parish bulletins, Catholic media, and personal contacts. Participants had to be churchgoing Catholics living in Australia, aged 18 years or over, and comfortable with reading English. Participation was voluntary and anonymous. The Monash University Human Research Ethics Committee approved the study (project number 25504).

Of the data set, 309 responses were excluded (20.2%) because they were incomplete and thus not submitted with final consent. An additional 44 responses were excluded because they fell below the defined threshold of ‘churchgoing Catholic’ (pre-closure Mass attendance rate of at least several times a month). Two responses were excluded because they did not provide Australian postcodes. The final data set comprised 1173 responses. For the current paper, this sample was further limited to participants who at the time (October 2020) had access to real-life worship in reopened churches, and who reported their post-church closure rate of Mass attendance (*N* = 806). All statistical analyses were carried out using IBM SPSS v27.

Participants were asked to provide self-reported five-year age range (18–24 to 80 +), self-identified gender which was recorded as 1 (*male*), 2 (*female*) and 3 (*other*), postcode, and country of birth to test sample representativeness against existing data on Australian churchgoing Catholics. They were also asked to indicate their pre- and post-church closure real-life Mass attendance rates (five options ranging from 0 = *Not at all* to 4 = *Sundays and some weekdays*).

Participants were also asked about their rates of engagement with virtual and real-life worship options during church closures (multiple options which were summed to produce a score representing the rate of engagement for both virtual and real-life worship for each participant). There were twelve real-life options presented, which included praying in another person’s home, attending a private Mass, and participating as technical staff at livestreamed Masses. There were thirteen virtual options presented, included watching livestreamed Masses, televised services, and video recordings of worship.

## Results

The final total sample (*N* = 806) was younger than the national average age for churchgoing Catholics, which may be why it also contained a higher proportion of Australian-born participants than the national average (Table 1). The state of Victoria had the most comprehensive church closure policy in Australia, so the sample for Victoria is not representative, as most Victorians could not attend Mass at the time the data were collected.

**Table 1:** Total Sample Compared to 2016 National Data on Australian Churchgoing Catholics

	Total N = 806	National Data^10^
Median age range (total range)	45 – 49 years (18 – 80 + years)	63 years
Gender (%)		
Male	337 (41.8%)	38.2%
Female	462 (57.3%)	61.8%
Other/no data	7 (0.9%)	−
Country of birth (%)		
Australia	624 (77.4%)	56.8%
Outside Australia	182 (22.6%)	43.2%
National distribution (%)		
NSW	354 (43.9%)	39.7%
VIC	56 (6.9%)	28.6%
QLD	135 (16.7%)	13.6%
WA	176 (21.8%)	10.9%
Other states and territories	85 (10.5%)	7.2%
Pre-closure Mass attendance rate (%)		
Several times a month	38 (4.7%)	−
Sundays only	195 (24.2%)	−
Sundays and some weekdays	573 (71.1%)	−

**Fig. 1:**
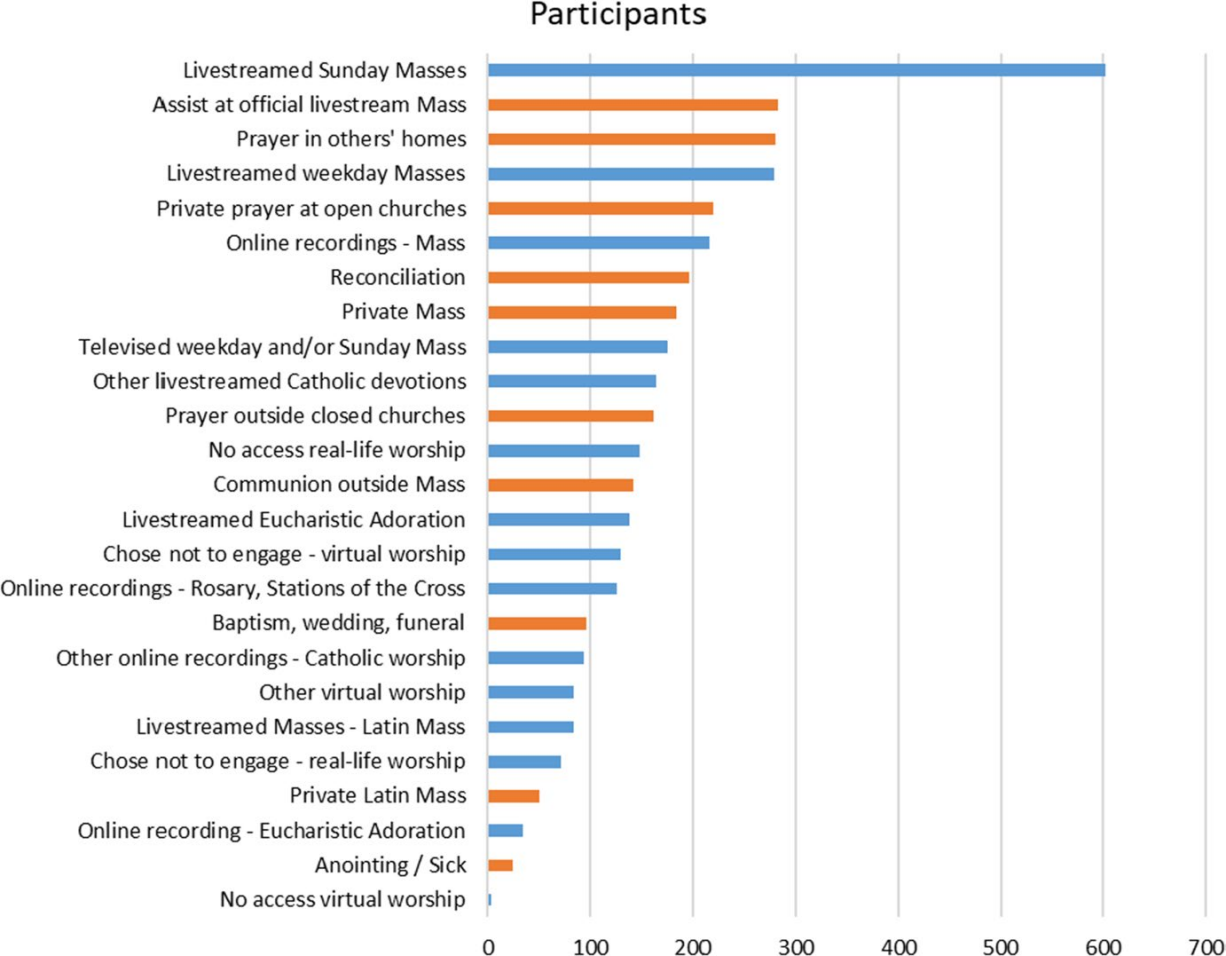
Real-life and virtual worship choices during COVID-19 church closures, by popularity (*N* = 806). *More than one choice was possible

The total sample were mostly highly engaged Catholics: over two-thirds of the total sample attended Mass very frequently (both Sundays and weekdays). The sample also captured roughly the same percentage of participants from every age group, from 18 to 80 + years. Most participants chose both virtual and real-life forms of worship during church closures—on average, around two forms of both virtual and real-life worship. Figure 1 shows the relative popularity of both real-life and virtual worship choices, with real-life choices indicated in orange.

To test Hypothesis 1—that rate of pre-closure Mass attendance is significantly and positively related to rates of real-life worship engagement during church closures—multiple regression analysis showed that age, gender, and pre-closure Mass attendance rate accounted for a significant 13% of the variability in rate of real-life worship engagement during church closures with a medium effect size, *R*^2^ = 0.13, adjusted *R*^2^ = 0.13, *F* (3,802) = 40.83, *p* < 0.001, *f*^2^ = 0.15. Unstandardised (B) and standardised (β) correlations (*sr*^2^) for each predictor in the regression model are shown in Table 2.

**Table 2:** Unstandardised (B) and Standardised (β) Regression Coefficients, and Squared Semi-Partial Correlations (sr^2^) for Each Predictor in a Regression Model Predicting Rate of Real-Life Worship Engagement

Variable	B [95% CI]	β	sr^2^
Age	−.11 [-.09, -.34]**	−.23	.05
Pre-closure Mass attendance rate	.84 [-.14, -.08]**	.27	.07
Gender	−.22 [-.43, -.01]*	−.07	.00

Note: n = 806. CI = confidence interval

**p ≤ .001 *p < 0.05

To test Hypothesis 1b—that rate of pre-closure Mass attendance is not significantly related to rate of engagement in virtual worship during church closures—multiple regression analysis showed that age, gender, and pre-closure Mass attendance rate accounted for a significant 8% of the variability in rate of virtual worship engagement during church closures with a small effect size, *R*^2^ = 0.08, adjusted *R*^2^ = 0.07, *F* (3,802) = 21.70, *p* < 0.001, *f*^2^ = 0.08. Unstandardised (B) and standardised (β) correlations (*sr*^2^) for each predictor in the regression model are shown in Table 3.

**Table 3:** Unstandardised (B) and Standardised (β) Regression Coefficients, and Squared Semi-Partial Correlations (sr^2^) for Each Predictor in a Regression Model Predicting Rate of Virtual Worship Engagement

Variable	B [95% CI]	β	sr^2^
Age	−.05 [−.08, −.02]*	−.10	.01
Pre-closure Mass attendance rate	.75 [.53, .96]**	.24	.05
Gender	.35 [.13, .57]*	.10	.00

Note: n = 806. CI = confidence interval

**p ≤ .001 *p < 0.05

To test Hypothesis 2—that rate of engagement in real-life worship during church closures is significantly and positively related to rate of return to Mass attendance after closures—multiple regression analysis showed that age, gender, pre-closure Mass attendance rate, rate of virtual worship engagement, and rate of real-life worship engagement accounted for a significant 25% of the variability in rate of return to Mass attendance with a large effect size, *R*^2^ = 0.25, adjusted *R*^2^ = 0.25, *F* (5,800) = 54.59, *p* < 0.001, *f*^2^ = 0.33. Unstandardised (B) and standardised (β) correlations (*sr*^2^) for each predictor in the regression model are shown in Table 4.

**Table 4:** Unstandardised (B) and Standardised (β) Regression Coefficients, and Squared Semi-Partial Correlations (sr^2^) for Each Predictor in a Regression Model Predicting Rate of Return to Mass Attendance

Variable		B [95% CI]	β	sr^2^
Age		.01 [-.01, .03]	.03	.00
Pre-closure Mass attendance rate		.76 [.64, .88]**	.39	.14
Gender		−11 [-.24, .01]	−05	.00
Virtual worship rate		.03 [-.01, .07]	.05	.00
Real-life worship rate		.12 [.08, .16]**	.20	.03

Note: n = 806. CI = confidence interval

**p ≤ .001 *p < 0.05

To test Hypothesis 3—that rate of engagement in real-life worship during church closures positively mediates the relationship between pre-closure and post-closure rates of Mass attendance—a mediation analysis was carried out in SPSS using the PROCESS 4.0 macro (Hayes, 2020). The mediation model accounted for significant unique variance in rate of post-closure Mass attendance, *R*^2^ = 0.25, *F* (2, 803) = 133.25, p < 0.001, with a Cohen’s *f*^2^ of 0.33, which can be considered a large effect size.

The model supported a direct effect of pre-closure Mass attendance rate predicting unique variance in the post-closure Mass attendance rate, holding the levels of real-life worship choice consistent across participants, *c’* = 0.77, *LLCI/ULCI* ≠ 0, *p* < 0.001. The model also supported an indirect effect (*ab*) of pre-closure Mass attendance rate via choice of real-life worship during church closures which accounted for significant unique variance in rate of post-closure Mass attendance, *ab* = 0.11, *LLCI/ULCI* ≠ 0, *p* < 0.001. Unstandardised (*B*) regression coefficients, 95% confidence intervals, and *R*^2^ values for the mediation model are presented in Table 5.

**Table 5:** Mediation Model Coefficients for Pre-Closure Mass Attendance Rate, Real-Life Worship During Closure, and Post-Closure Mass Attendance Rate (N = 806)

Variable	B [LLCI, ULCI]	SE
DV = Real-life worship choices (R^2^ = .08*)		
Constant	−93 [−2.91, −97]	.49
Pre-closure Mass attendance rate	.84 [.64, 1.05]	.10
DV = Post-closure Mass attendance rate (R^2^ = .25*)		
Constant	.35 [-.20, .90]	.28
Pre-closure Mass attendance rate	.77 [.66, .90]	.06
Real-life worship choices	.13 [.09, .17]	.02

*p < .001

To test whether there was a similar mediation effect for virtual worship choices, a second mediation model accounted for significant unique variance in rate of post-closure Mass attendance, *R*^2^ = 0.21, *F* (2, 803) = 112.04, p < 0.001, with a Cohen’s *f*^2^ of 0.26, which can be considered a medium effect size. This model also supported a direct effect of pre-closure Mass attendance rate predicting unique variance in the post-closure Mass attendance rate, holding the levels of real-life worship choice consistent across participants, *c’* = 0.84, *LLCI/ULCI* ≠ 0, *p* < 0.001. There was also an indirect effect (*ab*) of pre-closure Mass attendance rate via choice of virtual worship during church closures which accounted for significant unique variance in rate of post-closure Mass attendance, *ab* = 0.04, *LLCI/ULCI* ≠ 0, *p* < 0.001. Unstandardised (*B*) regression coefficients, 95% confidence intervals, and *R*^2^ values for the mediation model are presented in Table 6.

**Table 6:** Mediation Model Coefficients for Pre-Closure Mass Attendance Rate, Virtual Worship During Closure, and Post-Closure Mass Attendance Rate (N = 806)

Variable	B [LLCI, ULCI]	SE
DV = Virtual worship choices (R^2^ = .06*)		
Constant	−85 [-1.89, .15]	.51
Pre-closure Mass attendance rate	.75 [.53, .96]	.10
DV = Post-closure Mass attendance rate (R^2^ = .22*)		
Constant	.15 [-.40, .71]	.29
Pre-closure Mass attendance rate	.84 [.72, .97]	.06
Virtual worship choices	.06 [.02, 0.10]	.02

*p < .001

## Discussion

This study provides the first insight into how demographic and other factors might affect Australian Catholic worship choices in a situation involving mandated church closures for public health reasons. It provides a broad picture of how this group responded to government and diocesan restrictions on worship during COVID-19. It reveals willingness to engage in both real-life and virtual worship, but with a persistent preference for real-life worship even when churches are closed. The results have illuminated some of these complex relationships, which may help to assist both church and government planning for future management of church closures for this population.

Hypothesis 1a predicted that rates of pre-closure Mass attendance would be significantly and positively related to rate of engagement in real-life worship during church closures. This hypothesis was supported: all three predictors were significant with a medium effect size, but pre-closure Mass attendance rate showed the strongest positive relationship with rate of real-life worship during church closures.

Hypothesis 1b predicted that rate of pre-closure Mass attendance would not be significantly related to rate of engagement in virtual worship during church closures. This hypothesis was not supported: all three predictors were significant with a small effect size, and pre-closure Mass attendance rate was again the most strongly and positively related variable to rate of virtual worship during church closures. However, the effect size was smaller than that of Hypothesis 1a.

Hypothesis 2 predicted that rate of engagement in real-life worship during church closures would be significantly and positively related to rate of return to Mass attendance after closures. This hypothesis was supported: both pre-closure Mass attendance rate and real-life worship engagement rate were significantly and positively related to rates of return to Mass attendance after closures, with a large effect size. Virtual worship engagement rate did not show a significant relationship in this model.

Hypothesis 3—that the rate of engagement in real-life worship during church closures would positively mediate the relationship between pre-closure and post-closure rates of Mass attendance—was supported, with a large effect size. However, an identical mediation analysis testing the possible influence of virtual worship engagement on the same relationship also produced a statistically significant result, but with a smaller effect size.

These results confirm the importance of real-life worship for the Australian churchgoing Catholic population, and their strong attachment to this even during church closures. Overall, participants chose to engage virtual worship more frequently often than real-life worship, but it was the choice of real-life worship during church closures that appears to have had the strongest relationship with return to Mass attendance once churches reopened. Real-life worship engagement also strongly predicted return to Mass attendance after church closures, which shows that it has a sustaining and protective effect for Catholic churchgoers during difficult conditions such as the pandemic.

There appears to be a cascade-like relationship between these variables. The cascade begins with pre-closure Mass attendance rate, which was the strongest positive predictor of whether this population would choose real-life worship during church closures. This strong relationship was expected because of the in-person demands of Catholic worship, and because of the sudden mandatory change from real-life to virtual worship with little preparation time.

The choice for real-life worship then strongly influenced whether individuals would return to Mass attendance once churches reopened. This finding was expected because this population had high pre-closure Mass attendance rates. It corresponds with the small amount of literature available that indicates a consistent dissatisfaction with purely virtual worship in Christian communities (Hutchings, 2011; Wolff, 1999) and more recently an exceptionally low rate of expressed preference for exclusively virtual worship after churches reopen (CatholicVoices, 2020; Village et al., 2021).

By contrast, virtual worship showed a weaker predictive influence on return to Mass attendance. While a strong pre-closure Mass attendance history was significantly associated with choosing virtual worship engagement during church closures, virtual worship engagement did not then show a significant relationship to return to Mass attendance once churches reopened. The rate of virtual worship engagement also mediated the relationship between pre-closure Mass attendance rate and post-closure rate, but the effect was less strong than that of real-life worship engagement. The most protective effect on post-closure Mass attendance rates can be found in a high pre-closure rate of Mass attendance combined with a high rate of engagement in real-life worship during church closures.

Variables such as age and gender showed only limited predictive power. Age weakly and negatively predicted choice of both real-life and virtual worship, so that younger participants were more likely to engage in both than older participants. In Australia, those aged 60 years and over are more likely to be regular churchgoers (NCPR, 2020), so a positive relationship between increased age and choice of real-life worship was expected but did not appear. This could be because the sample was substantially younger (by around 20 years) than the national average for Australian churchgoing Catholics.

Gender showed similarly weak relationships, negatively predicting rate of real-life worship choices and positively predicting rate of virtual worship choices, meaning that males were more likely to choose real-life worship during church closures, and females more likely to choose virtual worship. The population in the current study was two-thirds female, which reflects the national gender distribution of Australian churchgoing Catholics.

Churchgoing has been associated in the literature with higher levels of personal wellbeing (Powell & Pepper, 2015). More epidemiological knowledge is also becoming available about the incidence and prevalence of COVID-19 and its transmission in vivo (Chang et al., 2021). Additionally, immunisation against COVID-19 is currently being rolled out nationally (Australia, 2021). It should now be possible for government and church leaders to adopt protocols for safer forms of real-life worship when outbreaks are experienced. Virtual worship may be safer from a public health perspective, but if Catholic Church leadership wishes to retain Mass-goers, it will need to argue that the loss of access to real-life worship for their community poses a risk to wellbeing which outweighs the risk of contagion (Baker et al., 2020).

A strength of this study is its exploratory approach. The situation of COVID-19 church closures is unprecedented in living memory in Australia and has provided a unique opportunity to explore questions around worship choices during mandated church closures. The enthusiastic national response to the survey is indicative of how seriously this population took the question of church closures and how strongly they felt the need to express their response to it. This study has also identified a population of churchgoing Catholics who are more likely to choose real-life worship even when churches are closed. Their data have provided a robust and credible picture of how this population responded to the loss of their normal forms of worship. It also makes this population easier to identify as in need of greater bridge-building with governments and with their own church leadership in future public health emergencies.

## Limitations

However, this strength is also possibly a weakness, as there may have been selection bias in the sample. The sample was already strongly oriented towards frequent real-life worship which may have affected their attitudes to its temporary suppression. The recruitment strategy was aimed largely at an online audience, who may also have been more comfortable with a virtual worship environment because of existing familiarity with the Catholic Internet world.

Demographic limitations may also affect the generalisability of these results. This sample is largely homogenous, and participation in this study was limited to those fluent in English, which could have excluded around a third of all churchgoing Catholics in Australia—the proportion born in countries where English is not a first language (NCPR, 2020). The high non-completion rate of surveys (around 20%) may be linked to this. The sample was also much younger than the national average age of churchgoing Catholics, which is a strength as well as a weakness: the results provide useful insights into the mindset of adult churchgoing Catholics aged under 50, who are more likely to continue churchgoing into the future, and thus to be affected by future pandemics and church closures. Finally, variations between individual state government restrictions on church attendance over the duration of the pandemic may also have affected responses.

This study was able to recruit a large nationwide sample quickly and with little difficulty, and the methodology could be replicated with additional questions. Multiple sociodemographic factors such as income, education level, and marital status also potentially affect rates of churchgoing. Any of these variables could also have affected the relationships and outcomes in this study and would be useful to consider in future research.

## Conclusion

There is ongoing interest in expanding virtual forms of worship in the Catholic Church (Rocca, 2020). It may be possible to identify Catholic sub-populations for whom virtual worship would be preferred and optimal. Comparing Catholic data to similar data from other collectively and publicly worshipping religious traditions, some of whom also chose to engage in real-life worship during closures, may also reveal different relationships with the resumption of normal real-life worship attendance.

The COVID-19 pandemic has stimulated interest in the relationships between religion, spirituality, and health, and churchgoing has been linked positively to overall wellbeing. Virtual worship has a place in supporting Catholics during church closures, but it does not provide the same impetus towards return to Mass attendance as real-life worship engagement. In future outbreaks and pandemics, limited forms of real-life worship with social distancing and PPE should be possible. These protocols could be adapted so that other worship communities could meet their real-life worship needs safely and sustain healthy real-life restoration of those communities after a pandemic.
